# UV–Vis spectroscopy of tyrosine side-groups in studies of protein structure. Part 2: selected applications

**DOI:** 10.1007/s12551-016-0197-7

**Published:** 2016-05-04

**Authors:** Jan M. Antosiewicz, David Shugar

**Affiliations:** 1 Division of Biophysics, Institute of Experimental Physics, Faculty of Physics, University of Warsaw, żwirki i Wigury 93, 02-089 Warsaw, Poland; 2 Institute of Biochemistry & Biophysics, Polish Academy of Sciences, Pawinskiego 5a, 02-106 Warsaw, Poland

**Keywords:** Tyrosine, UV–Vis absorption, Fluorescence, Linear and circular dichroism, Resonance Raman scattering

## Abstract

In Part 2 we discuss application of several different types of UV–Vis spectroscopy, such as normal, difference, and second-derivative UV absorption spectroscopy, fluorescence spectroscopy, linear and circular dichroism spectroscopy, and Raman spectroscopy, of the side-chain of tyrosine residues in different molecular environments. We review the ways these spectroscopies can be used to probe complex protein structures.

## Introduction

The UV absorption of proteins in the range 180 to 230 nm is due almost entirely to $\pi \rightarrow \pi ^{*}$ transitions in the peptide bonds. Absorption in the range of 230–300 nm is dominated by the aromatic side-chains of tryptophan (Trp), tyrosine (Tyr), and phenylalanine (Phe) residues, and there is weak contribution by disulphide bonds near 260 nm (Goldfarb et al. 1951; Aitken and Learmonth 2009; Fornander et al. 2014).

Employment of optical properties of Tyr residues in structural studies of proteins began with a report on the differences in the state of tyrosine in native and denatured proteins, shown by the pH-dependence of the absorption spectrum, as described by Crammer and Neuberger (1943). This was followed by our report that used UV absorption to show that ribonuclease contains three (of six) abnormal tyrosyl residues that ionize only after denaturation at elevated pH (Shugar 1952).

In a report by Crammer and Neuberger on Tyr ionization in egg albumin and insulin (Crammer and Neuberger 1943), the authors used spectrophotometric titration to probe reactions between the protein and hydrogen and hydroxyl ions to characterize ionizable groups in these proteins. Interpretation of the titration curves is often ambiguous, because of the overlap of the titration ranges of different ionizable groups in proteins. Facing these difficulties, Crammer and Neuberger wrote: “*It occurred to us that the ionization of the phenolic group of tyrosine in the protein might be investigated by a spectroscopic method, without interference from other groups ionizing in the same region*”. They concluded that changes of the absorption spectrum of egg albumin with pH indicate that Tyr residues in the native protein are not free to ionize, due to restrictions imposed upon the protein configuration by its tertiary structure. Following denaturation, ionization could be demonstrated spectroscopically (Crammer and Neuberger 1943). Similar conclusions were reached by Shugar (1952) using ribonuclease. Subsequently, anomalous Tyr ionization in proteins, detected by spectrophotometric titration, and other properties of Tyr, have become generally accepted as reflections of secondary and/or tertiary structures of the proteins (Gorbunoff 1967).

Here we present selected examples of application of conventional, difference, and second-derivative UV absorption spectroscopy, fluorescence spectroscopy, linear and circular dichroism spectroscopy and Raman spectroscopy of the side-chain of Tyr residues to investigate different aspects of protein structure. Together, they help us analyze not only their secondary and tertiary structures but also the role of solvent accessibility of Tyr chromophores in hydrogen bonding and/or hydrophobic interactions, pK_*a*_s of the Tyr hydroxyl, and dependence of these parameters on factors such as solvent and binding of ligands.

## Solvent exposure and ionization of tyrosine side-groups in proteins studied by UV absorbance spectroscopy

### Spectrophotometric titration of tyrosines

**Fig. 1:**
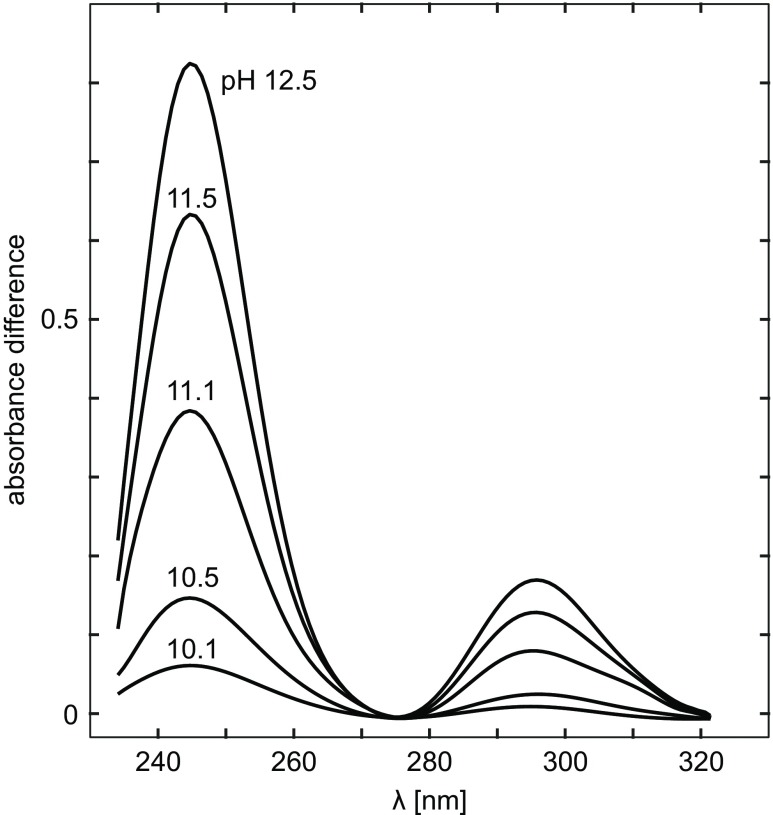
The difference spectra of actin as a function of pH. Protein concentration is 0.17 mg/ml. The reference solution is of the same protein concentration at pH 7.3. Taken from Mihashi and Ooi (Mihashi and Ooi 1965)

Crammer and Neuberger (1943) and Shugar (1952) were the first to use UV–Vis spectroscopy to study the optical properties of the Tyr chromophore and interpret changes in absorbance spectra of proteins as a function of pH. These studies were based on observation of the ionization of Tyr hydroxyl that results in significant changes in the phenolic absorption spectrum, including a red shift of the major absorbance peaks from 222 and 275 nm to 242 and 295 nm, respectively, and significant hyperchromic effects with both peaks (Kueltzo and Middaugh 2005). These changes are clearly visible in the pH difference spectrum for the ionization of Tyr, which has two maxima, at 295 nm (Crammer and Neuberger 1943; Shugar 1952), and 245 nm (Hermans 1962). Figure 1 presents difference spectra of actin as a function of pH, with the spectrum at pH 7 as the reference, reported by Mihashi and Ooi (1965). Actin is a muscle protein that exists in a globular form (G-actin) in low-salt solutions, and polymerizes into long fibrous molecule (F-actin) when neutral salt is added.

Changes in absorption spectra, followed at ∼295 and/or ∼245 nm, indicate either ionization of exposed Tyr side-groups, when they occur at lower pH range, or ionization of buried residues at pH values sufficiently high to cause denaturation of the protein and exposure of Tyr residues buried before the transition. As a reference value of the pK_*a*_, which allows us to consider it as high or normal, we can take a value found for the Tyr chromophore fully exposed to the solvent, e.g., 9.76 for acetyl-Gly-Tyr-Gly-amide by NMR spectroscopy (Platzer et al. 2014).

Spectrophotometric titration can provide more specific structural information. Mihashi and Ooi (1965) noted that abnormal Tyr residues remain even in 6 M urea, even though such conditions unfolded helical segments of the protein. They concluded that the helical segments are located near the surface and Tyr located at these segments represent one kind of buried residues. Simultaneously, deeply buried Tyr interact with other side-chain groups to maintain a rigid structure, presumably forming the core of the actin monomer. Urea destroys the helical structure of the molecule but is not strong enough to disrupt the interaction of deeply buried Tyr with other groups in the core. Addition of guanidine-HCl unfolds not only of the helical region but also of the region where the more stable abnormal Tyr residues are buried, perhaps consisting of hydrophobic amino acid residues. Classification of the abnormal Tyr into two kinds is consistent with this. Furthermore, the region containing the abnormal Tyr is distinguishable from a region that was critical for polymerization, since the loss of polymerizability occurs before normalization of the Tyr by guanidine-HCl.

These titrations can be combined with crystallography, as e.g., for vipoxin neurotoxic phospholipase A_2_ (PLA_2_) (Georgieva et al. 1999). This basic protein has a single polypeptide chain containing 122 residues, including eight Tyr residues. Earlier crystallographic studies resulted in a three-dimensional structure, and allowed the authors to determine solvent accessibility of the Tyr residues. Then, by spectrophotometric titration, they found three Tyr with a pK_eff_ = 10.45, three with a pK_eff_ = 12.17, and two with a pK_eff_ = 13.23. Based on solvent accessibilities, Y117, Y75, and Y22 were identified as the first group, Y28, Y113, and Y52 as the second group, and Y25 and Y73 as the last group.

### Derivative absorption spectroscopy

**Fig. 2:**
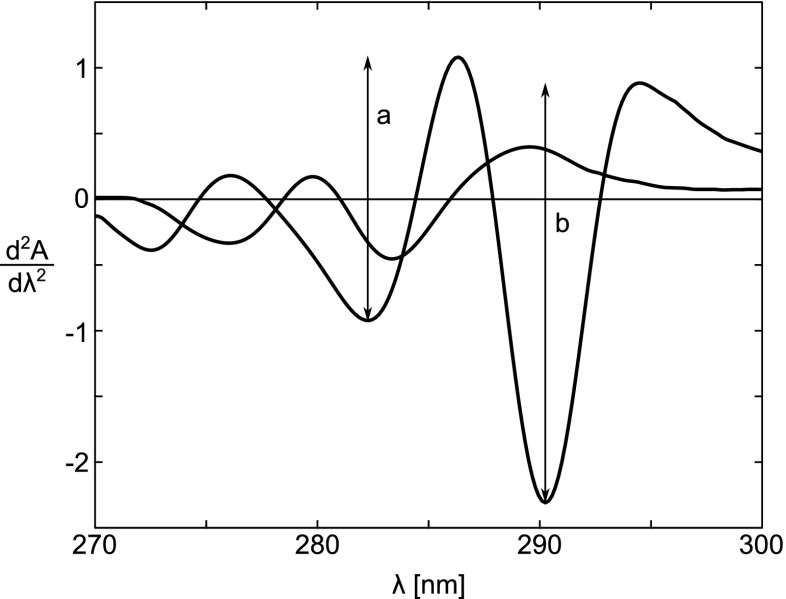
Second-derivative spectra of equimolar solutions of N-Ac-Trp-NH_2_ and N-Ac-Tyr-NH_2_ dissolved in 6.0 M Gdn ⋅HC–0.05 M phosphate, pH 6.5. The spectrum of N-Ac-Trp-NH_2_ is identified by the two *arrowsa* and *b*, which indicate the peak-to-peak distances between the maximum at 287 nm and the minimum at 283 nm, and the maximum at 295 nm and the minimum at 290.5 nm, respectively. Taken from Ragone et al. (1984)

Determination of Tyr exposure in proteins by second-derivative spectroscopy was proposed by Ragone et al. (1984), based on an earlier study of Servillo et al. (1982). This simple method examined Tyr and Trp in native proteins. It is based on the observation that the second-derivative spectrum of N-Ac-Trp-NH_2_, between 280 and 300 nm (see Fig. 2) shows two maxima centered at 287 and 295 nm and two minima at 283 and 290.5 nm (Servillo et al. 1982; Ragone et al. 1984), and that they are only marginally affected by solvent polarity. In the same spectral region, N-Ac-Tyr-NH_2_ exhibits a single minimum centered at 283.5 nm and two maxima at 280 and 289.5 nm. Because intensities of Trp bands are much higher than those of Tyr bands, the second-derivative spectra of samples containing both more closely resemble that of N-Ac-Trp-NH_2_, even in the presence of high Tyr concentrations (Servillo et al. 1982).

Ragone et al. (1984) focused on the ratio between two peak-to-peak distances, *r*_*p*_≡*a*/*b*, with *a* and *b* defined in Fig. 2, in the second-derivative spectrum of N-Ac-Trp-NH_2_

1 $$ r_{p} \equiv \frac{a}{b} = \frac{A^{\prime\prime}(\mathrm{287nm})-A^{\prime\prime}(\mathrm{283nm})}{A^{\prime\prime}(\mathrm{295nm})-A^{\prime\prime}(\mathrm{290.5nm})} $$

They found that *r*_*p*_ is almost the same over a wide range of solvent polarities, with a mean value of 0.68 ±0.02. Considering the absorbance at any given wavelength of a mixture of the Trp and Tyr chromophores,

$$A = \epsilon_{\text{Trp}} \cdot C_{\text{Trp}} + \epsilon_{\text{Tyr}} \cdot C_{\text{Tyr}} $$

they derived the following equation for the second-derivative differences Δ*A*_1_≡*A*_287_−*A*_283_ and Δ*A*_2_≡*A*_295_−*A*_290.5_:

2 $$ \frac{\Delta A_{1}^{\prime\prime}}{\Delta A_{2}^{\prime\prime}} = \frac{\frac{\Delta\epsilon^{\prime\prime}_{1,\,\text{Trp}}}{\Delta\epsilon^{\prime\prime}_{2,\,\text{Trp}}} + \frac{\Delta\epsilon^{\prime\prime}_{1,\,\text{Tyr}}}{\Delta\epsilon^{\prime\prime}_{2,\,\text{Trp}}} \cdot \frac{C_{\text{Tyr}}}{C_{\text{Trp}}}}{1 + \frac{\Delta\epsilon^{\prime\prime}_{2,\,\text{Tyr}}}{\Delta\epsilon^{\prime\prime}_{2,\,\text{Trp}}} \cdot \frac{C_{\text{Tyr}}}{C_{\text{Trp}}}} \equiv \frac{{\mathcal{A}} x + {\mathcal{B}}}{{\mathcal{C}} x + 1} \equiv r_{d} $$

where *x*≡*C*_Tyr_/*C*_Trp_, and definition of coefficients ${\mathcal {A}}$, ${\mathcal {B}}$, ${\mathcal {C}}$ clearly results from comparison of the two right terms in Eq. 2. Finally, in analogy to Eq. 1, defining *r*_*p*_, Eq. 2 may be considered as a definition of a similar quantity, given the symbol *r*_*d*_. Note that if *C*_Tyr_ = 0, then *r*_*d*_ = *r*_*p*_, and the right-hand side of Eq. 2 can be written as

3 $$ r_{d} = \frac{{\mathcal{A}} x + r_{p}}{{\mathcal{C}} x + 1} $$

which is a useful form if we take into account the experimentally verified independence of *r*_*p*_ on solvent composition. However, Ragone et al. (1984) found that *r*_*d*_ depends not only on the molar ratio *x* between Tyr and Trp, but also on the solvent composition. This dependence is a consequence of the fact that the numerical values of coefficients $\mathcal {A}$ and $\mathcal {C}$ depend on the properties of the solvent. Investigating these dependencies, they showed that the numerical value of *r*_*d*_ for a given Tyr/Trp ratio is determined by the molecular environment of tyrosine.

The second-derivative spectrum of proteins containing both Tyr and Trp residues shows the same general features observed for mixtures of N-Ac-Tyr-NH_2_ and N-Ac-Trp-NH_2_, i.e., two minima centered around 283 and 290.5 nm and two maxima around 287 and 295 nm. Since the position of the peaks do not change much after exposure to perturbing agents, Ragone et al. (1984) analyzed the second-derivative spectra of proteins in terms of the ratio between the peak-to-peak distances *a* and *b*. They proposed the following equation to determine the degree of Tyr residue exposure to solvent in native proteins:

4 $$ \alpha = \frac{r_{n} - r_{a}}{r_{u} - r_{a}} $$

where *r*_*n*_ and *r*_*u*_ are the numerical values of the ratio *a*/*b* determined for the native and unfolded protein, respectively, and *r*_*a*_ is the *a*/*b* value of a mixture, containing the same molar ratio of aromatic amino acids dissolved in a solvent with the same characteristics as the interior of the protein, e.g. ethylene glycol. The fraction of Tyr residues exposed to solvent in several proteins with native structure, determined by second-derivative measurements, appears to be in good agreement with those available from crystallography or other methods (Ragone et al. 1984).

**Fig. 3:**
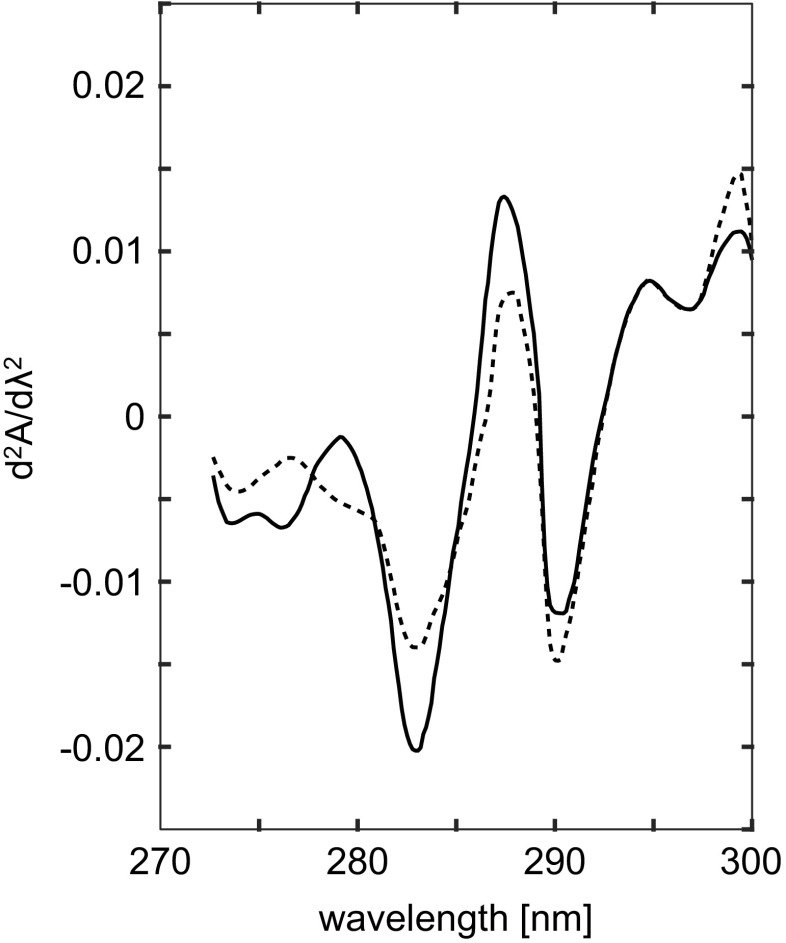
Isosbestic point at ∼284.2 nm in the second-derivative spectra of 2.3 *μ*M native (50 mM Tris/acetate buffer pH 7.4; *full line*) and Gdn ⋅HCl denatured MM-creatine kinase (50 mM Tris/acetate, 6 M Gdn,HCl buffer pH 7.4; *dotted line*). Adapted from Leydier et al. (1997)

**Fig. 4:**
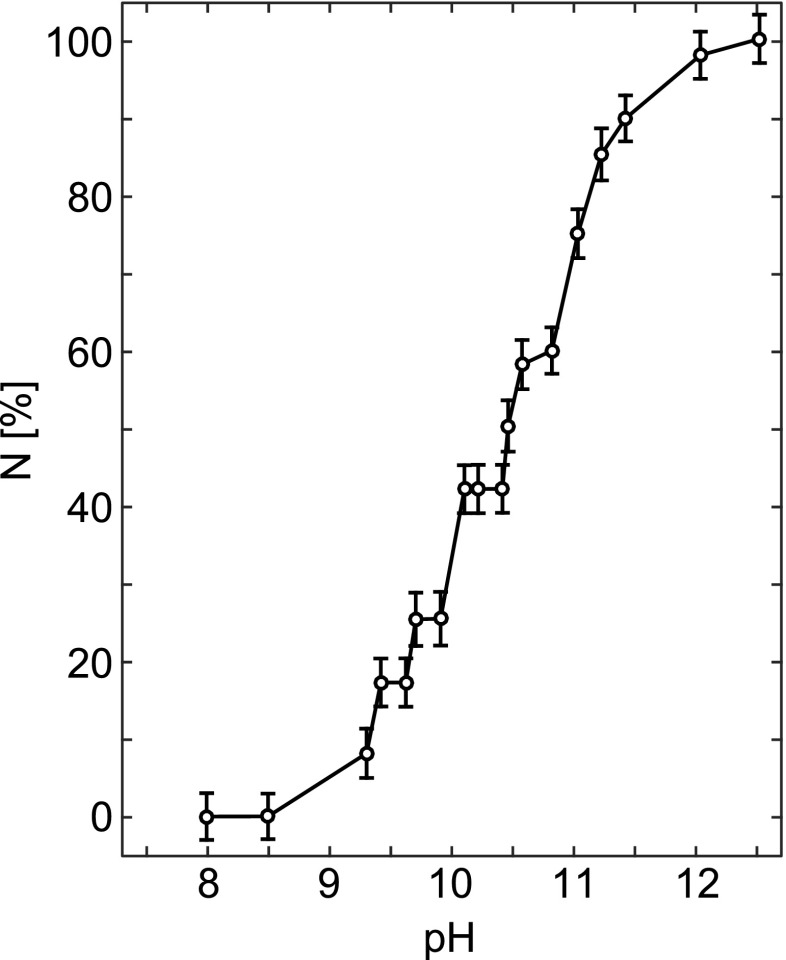
Titration curve of the Tyr residues in Carlsberg subtilisin obtained by the difference SDUV method, shown as % of ionized Tyr residues, N, vs. pH. Adapted from Breydo et al. (1997)

Second-derivative UV absorbance spectroscopy may be also used to determine pK_*a*_ of Tyr side-chains in proteins (Breydo et al. 1997). The method monitors the decrease in intensity of the second-derivative of the spectrum at the isosbestic point corresponding to the transition between buried and exposed non-ionized Tyr residues (284.2 nm). Existence of the isosbestic point (see an example in Fig. 3) indicates that the transition from buried to exposed states of Tyr is a transition between two discrete conformations of the protein. Hence, changes in the second-derivative of the UV (SDUV) absorbance spectrum for a protein, at 284.2 nm, as a function of pH, reflects changes in the ionization state of the protein Tyr residues. An example of such titration curve is shown in Fig. 4.

A modification of the above method was introduced for proteins with high levels of Trp residues (Tyr:Trp ratio less than 2, e.g., for chymotrypsinogen and chymotrypsin). Breydo et al. (1997) analyzed such cases by using difference SDUV, i.e., by subtracting the spectrum of the same protein at the same concentration and at neutral pH from the spectrum recorded at a current pH.

The development of high-resolution ultraviolet absorbance spectroscopy permits the detection of the position of derivative peaks with a resolution approaching 0.01 nm. This encouraged (Lucas et al. 2006) to use the technique to investigate cation- *π* interactions in proteins. Their approach used high-chloride salt concentrations (Li ^+^, Na ^+^, and Cs ^+^), chosen on the basis of their ionic radii to drive the diffusion of cations into interior regions of proteins where the cations interact with the aromatic amino acid side-chains (Phe, Trp, Tyr). For each aromatic amino acid, they selected a peak in the SDUV spectrum to observe shifts as a function of salt concentration. They expected to deduce local structure and dynamics of the eight selected proteins, albeit with varying degrees of exposure of particular side-chains. As control experiments, they investigated the ability of the three cations to shift the positions of aromatic absorbance peaks of the *N*-acetylated C-ethyl esterified amino acids, representing totally exposed side-chains. Here we focus on their results for Tyr residues.

**Fig. 5:**
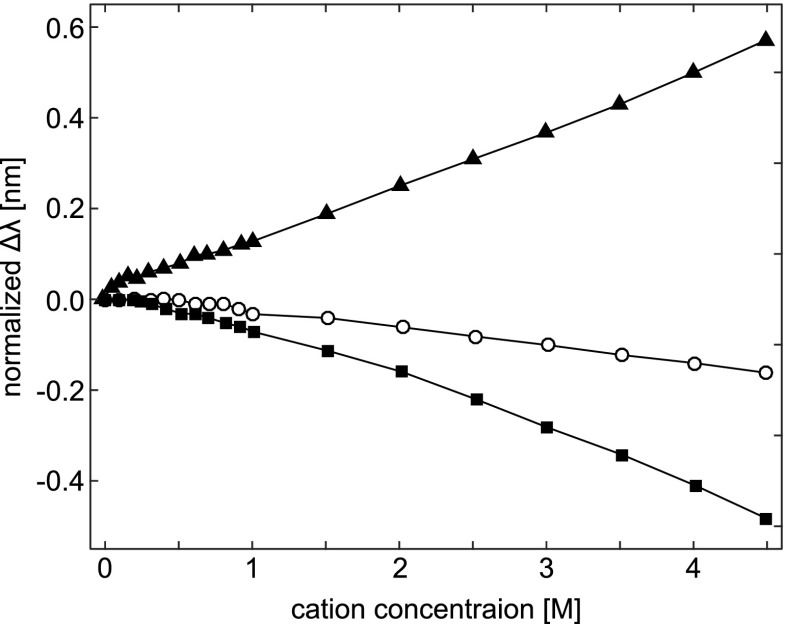
Peak shifts (Δλ) in the second-derivative UV absorption spectra for the model amino acid, *N*-acetyl-l-tyrosine ethyl ester, induced by the cations Li ^+^ (*filled squares*), Na ^+^ (*open circles*), and Cs ^+^ (*filled triangles*). The peak positions at each concentration point are normalized by subtracting the initial peak position, which was 275.01 ±0.01 nm. Taken from Lucas et al. (2006)

Figure 5 shows the relative peak shifts for *N*-acetylated carboxyl ethyl ester of Tyr as a function of cation type and concentration. Amino acids *N*-acetylated and esterified at the C-termini were chosen to minimize electrostatic interactions that could complicate interpretation of spectral changes due to cation- *π* effects between the salts and the aromatic ring of an amino acid. The cations appear to induce quite dramatic peak shifts in the Tyr spectra. Cs ^+^ causes a large red (positive) shift, Na ^+^ a small blue (negative) shift, while Li ^+^ causes a large negative change, approximately equal in magnitude to the red shift effected by Cs ^+^. These differences are also seen at concentrations of the order of 0.25 M, suggesting that Tyr peak position is sensitive to both cation size or charge density and concentration.

**Fig. 6:**
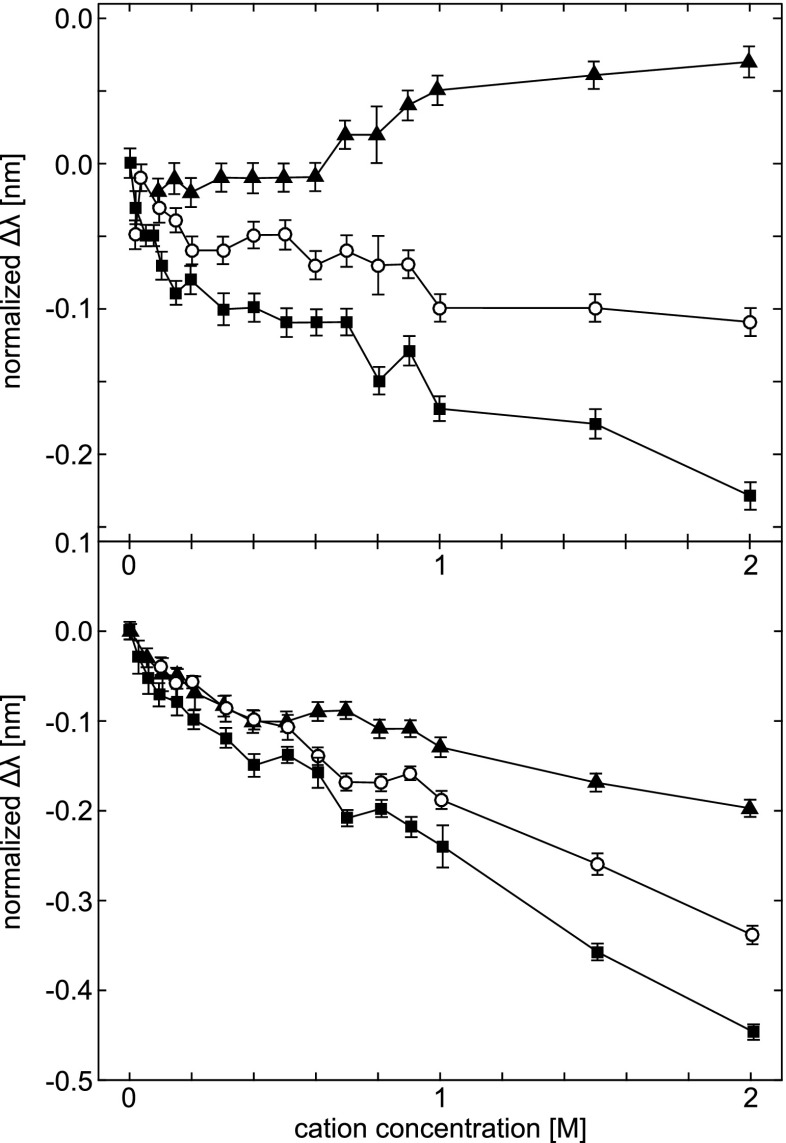
Tyr peak shifts in the second-derivative UV absorption spectra of Ribonuclease T1 (*top*) and human serum albumin (*bottom*), induced by the cations Li ^+^ (*filled squares*), Na ^+^ (*open circles*), and Cs ^+^ (*filled triangles*). The data are displayed as in Fig. 5, with initial peak positions of 277.61 ±0.01 nm for RNAse T1, and 278.82 ± 0.01 nm for HSA. *Error bars* represent the standard deviation of the mean for three experiments for each salt. Taken from Lucas et al. (2006)

Figure 6 illustrates the Tyr peak shift data for ribonuclease T1 (RNase T1, top), and human serum albumin (HSA, bottom), respectively. Only these two of eight proteins investigated by Lucas et al. (2006) are shown. The peak shifts for RNase T1 are similar to those for the free amino acid analog (Fig. 5). Increasing the Cs ^+^ concentration causes a red shift of the Tyr peak, while Na ^+^ causes an intermediate change, and Li ^+^ a blue shift. Although the shifts are small, the similarity of these data to that of *N*-acetyl-l-tyrosine ethyl ester suggests that one or more of the protein Tyr residues are located near the surface. The average solvent-accessible surface area for the Tyr residues of RNase T1 is relatively low, but the range of areas reveals that indeed some of the Tyr residues are near the protein surface.

The peak shifts for HSA (top of Fig. 6) are similar to those for the free amino acid analog (Fig. 5) in the case of Li ^+^ and Na ^+^ cations, whereas for Cs ^+^ trends in the second-derivative UV spectra are contrary to the control data for free Tyr. This suggests that the phenyl rings are buried, or that the protein structure has been perturbed. The three cations induce a large blue shift in the HSA Tyr peak (bottom of Fig. 6). The authors concluded these changes indicate that hydrogen bonding dominates the observed peak shifts in the presence of all three cations. The Tyr residues of this protein have a relatively high degree of solvent exposure compared to Tyr residues in other proteins studied by them.

### Kinetic aspects of Tyr exposure and/or pK_*a*_ determination, revealed by stopped-flow spectrometry

Kuwajima et al. (1979) developed a stopped-flow technique for zero-time spectrophotometric titration of Tyr residues in the native or in the completely alkaline-denatured state applicable to proteins that undergo relatively fast alkaline conformational transition in the pH region of Tyr ionization.

When pH of a protein solution is suddenly raised sufficiently high in a stopped-flow experiment, two kinds of changes occur: (1) Accessible Tyr are ionized (this process is completed within dead time of stopped-flow apparatus, i.e., ∼1 ms); and (2) A reversible time-dependent change in conformation (which can make other Tyr accessible, and then ionized) is induced, which proceeds to an equilibrium endpoint that, in turn, depends on pH. If these changes are observed using UV absorption, the total change can be represented as a sum of three contributions (Kuwajima et al. 1979)

5 $$ {\Delta} A = {\Delta} A_{\text{ion}} + {\Delta} A_{\text{conf}} + {\Delta} A_{\mathrm{conf+ion}} $$

One can only observe contributions of the second and third term in the right-hand side of Eq. 5 to the time-dependent absorbance changes, Δ*A*_conf_ and Δ*A*_conf+ion_, provided that the conformational change caused by the pH-jump is not too rapid for stopped-flow experiments. When this condition is satisfied, then extrapolation of the observed absorption changes to zero time gives information about the ionization equilibrium in the native state at the alkaline pH. Determining this absorbance change relative to zero time as a function of the final alkaline pH leads to a titration curve, which was denoted by Kuwajima et al. (1979) as the zero-time titration curve for the native state. Similar analysis for pH-jumps from highly alkaline pH to lower values resulted in the zero-time titration curve for the purely alkaline-denatured protein.

Kuwajima et al. (1979) applied this to bovine *α*-lactalbumin, characterized by occurrence of fast alkaline denaturation, which makes determination of the ionization behavior of tyrosines by usual spectrophotometric techniques practically impossible.

First, it was necessary to separate contributions to the spectra due to ionization of the Tyr residues from contributions due to alkaline denaturation itself. To obtain the spectrum caused by alkaline denaturation, Kuwajima et al. carried out pH-jump experiments from pH 11.6 to pH 8.0. At pH 8.0 the protein is in the native (N) state and all its Tyr residues are protonated. At the initial pH of 11.6, the protein is in the alkaline-denatured (D) state. With a pH-jump from 11.6 to 8.0 in a stopped-flow spectrometer, protonation of the Tyr residues exposed to the solvent at the starting pH, is instantaneously lost in the dead time of the experiment leaving only the absorbance change corresponding to Δ*A*_conf_ in the kinetic traces. This allowed Kuwajima et al. to determine kinetic amplitudes observed by the pH-jump from 11.6 to 8.0, as a function of the incident wavelength. They showed that at 298 nm the difference absorption in the spectrum is essentially zero. Thus, the absorption change at 298 nm can be taken as a measure of Tyr ionization.

**Fig. 7:**
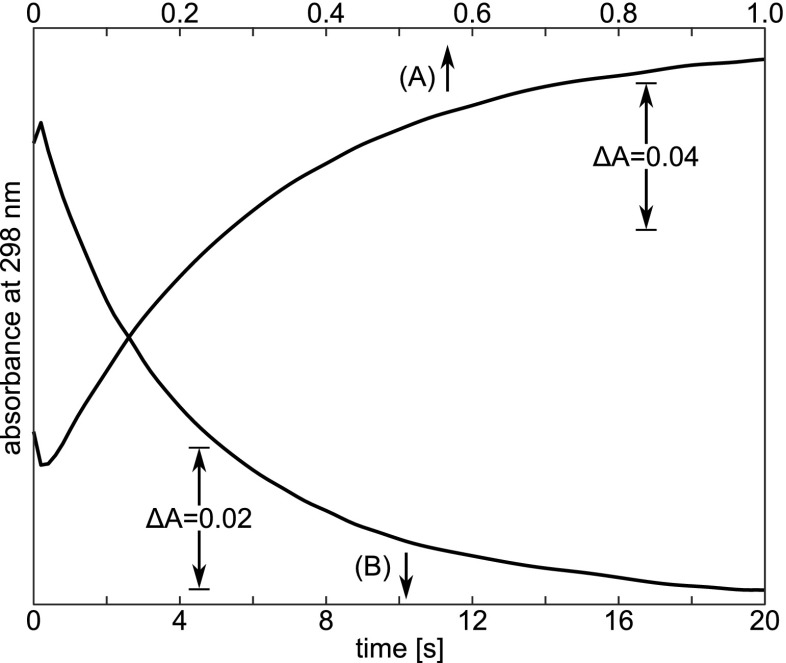
Typical time-courses of the absorption changes at 298 nm after a pH-jump at 0.5 M Gdm-HCl and 25.0 ^∘^C : **a** forward pH-jump from pH 5.5 to 11.3; and **b** reversed pH-jump from 11.8 to 10.2. Protein concentration is ca. 0.05 %. Taken from Kuwajima et al. (1979)

Figure 7 shows the typical kinetic traces for absorption at 298 nm from stopped-flow experiments, one for a pH-jump from pH 5.5 to 11.3, and the other from pH 11.8 to 10.2. These were analyzed using a first-order rate law:

6 $$ A(t)-A_{o} = \left( A_{\infty} - A_{o}\right) \cdot \left( 1-\exp(-kt)\right) $$

where *A*(*t*) is the absorption at time *t* after the pH-jump, $A_{\infty }$ and *A*_*o*_ are the values at infinite and at zero time, respectively, and *k* is the first-order rate constant. The difference $A_{\infty }-A_{o}$ represents the desired “kinetic amplitude” of the observed processes (${\Delta }\epsilon _{\mathrm {298}}^{f}$ for forward, and ${\Delta }\epsilon _{\mathrm {298}}^{r}$ for reversed pH-jump, respectively).

The pH-dependence of the equilibrium difference extinction coefficient at 298 nm, ${\Delta }\epsilon _{\mathrm {298}}^{\text {eq}}$, gives the equilibrium ionization curve of the alkaline denaturation. The transition from low pH (where the structure is native) to high pH (fully denatured state) exhibits a transition zone when both native and alkaline denatured species participate to the ionization equilibrium. Assuming a two-state transition, ${\Delta }\epsilon _{\mathrm {298}}^{\text {eq}}$ can be related to the fractions of the native, (*f*_*N*_), and denatured, (*f*_*D*_), species, and the degrees of Tyr ionization in both species, (*α*_N, i_ and *α*_D, i_) as expressed by the following:

7 $$ {\Delta}\epsilon_{\mathrm{298}}^{\text{eq}} = {\Delta}\epsilon_{\mathrm{298}}^{\mathrm{0}} \sum\limits_{i=1}^{n} \left( f_{N} \alpha_{\mathrm{N,\,i}} + f_{D} \alpha_{\mathrm{D,\,i}}\right) $$

where ${\Delta }\epsilon _{\mathrm {298}}^{\mathrm {0}}$ is the extinction coefficient for ionization of a single Tyr residue, and *n* is the number of Tyr residues in the protein (four in the case of *α*-lactalbumin).

**Fig. 8:**
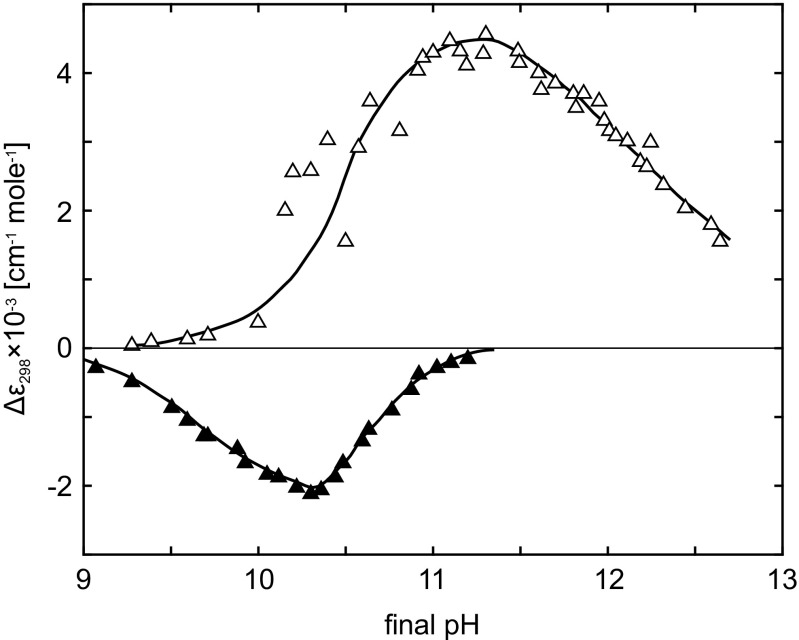
Dependence of ${\Delta }\epsilon _{\mathrm {298}}^{f}$ (*empty triangles*) and ${\Delta }\epsilon _{\mathrm {298}}^{r}$ (*full triangles*) on final pH. Taken from Kuwajima et al. (1979)

When *α*_N, i_ differs significantly from *α*_D, i_ at the final pH in pH-jump experiments, the conformational change caused by the pH-jump leads to a change in Tyr ionization. This can be observed as a time-dependent absorption change of Δ*A*_conf+ion_ at 298 nm, following a pH-jump from an initial value (∼5.5 or ∼11.8) to some final pH, obeying the alkaline-transition region. Resulting amplitudes, ${\Delta }\epsilon _{\mathrm {298}}^{f}$ and ${\Delta }\epsilon _{\mathrm {298}}^{r}$, plotted against the final pH are shown in Fig. 8. The bell-shaped features of the plots are expressed by:

8 $$ {\Delta}\epsilon_{\mathrm{298}}^{f} = {\Delta}\epsilon_{\mathrm{298}}^{0} f_{D} \sum\limits_{i=1}^{4} \left( \alpha_{\mathrm{D,\,i}} - \alpha_{\mathrm{N,\,i}}\right) $$

and

9 $$ {\Delta}\epsilon_{298}^{r} = {\Delta}\epsilon_{298}^{0} f_{N} \sum\limits_{i=1}^{4} \left( \alpha_{\mathrm{N,\,i}} - \alpha_{\mathrm{D,\,i}}\right) $$

where *f*_*N*_, *f*_*D*_, *α*_N, i_, and *α*_D, i_ refer to the final pH. From Eqs. 7–9, and the condition *f*_*N*_ + *f*_*D*_ = 1, one obtains

10 $$ {\Delta}\epsilon_{298}^{\text{eq}} - {\Delta}\epsilon_{298}^{f} = {\Delta}\epsilon_{298}^{0} \sum\limits_{i=1}^{4} \alpha_{\mathrm{N,\,i}} $$

and

11 $$ {\Delta}\epsilon_{298}^{\text{eq}} - {\Delta}\epsilon_{298}^{r} = {\Delta}\epsilon_{298}^{0} \sum\limits_{i=1}^{4} \alpha_{\mathrm{D,\,i}} $$

These two quantities correspond to the zero-time difference extinction coefficients of Tyr ionization after the pH-jump. They do not include any contributions from the conformational transition. Thus, they can be used to plot the zero-time titration curves of tyrosines in the purely N and the purely D states, respectively.

Assuming independent ionization of the tyrosines, justified by the relatively high ionic strength of the solutions, the degrees of Tyr ionization can be related to equilibrium ionization constants *K*_N, i_ and *K*_D, i_, respectively and hydrogen ion activity $a_{H^{+}}$:

12 $$ \alpha_{\mathrm{N,\,i}} = \frac{K_{\mathrm{N,\,i}}}{K_{\mathrm{N,\,i}}+a_{H^{+}}} \quad \text{and} \qquad \alpha_{\mathrm{D,\,i}} = \frac{K_{\mathrm{D,\,i}}}{K_{\mathrm{D,\,i}}+a_{H^{+}}} $$

These last expressions can be used in Eqs. 10 and 11 to fit theoretical predictions to experimental observations, resulting in values of equilibrium ionization constants defined by Eq. 12. The values of ${\Delta }\epsilon _{\mathrm {298}}^{0}$ were estimated from ${\Delta }\epsilon _{\mathrm {298}}^{0}$ at pH 12, where all the Tyr are fully ionized, since ${\Delta }\epsilon _{\mathrm {298}}^{0}$ of the protein completely denatured by 6 M Gdn ⋅HCl is almost the same as that for 0.5 M Gdn ⋅HCl at pH 12.

The result is that, in the denatured state, all Tyr have pK_*a*_ equal to 10.3, whereas in the native state they are: pK_*a*, Tyr18_ = 11.8, pK_*a*, Tyr36_ = 11.8, pK_*a*, Tyr50_ = 12.7, and pK_*a*, Tyr103_ = 10.5 (Kuwajima et al. 1979).

## Protein structure changes measured by tyrosine fluorescence

Tyr exhibits substantial fluorescence, and the high environmental sensitivity of its emission, making it a useful natural probe for studying structure and dynamics of proteins. Intrinsic fluorescence of proteins also results from excitation of two other aromatic amino acids, Trp and Phe. Because of the dominant emission of Trp, fluorescence of Tyr can normally be routinely observed in proteins that lack Trp residues (Lakowicz et al. 1995). However, as noted by Edelhoch et al. (1969), in some proteins, fluorescence of both Trp and Tyr can be observed simultaneously. One such protein is the parathyroid hormone, PTH, an important endocrine regulator of calcium and phosphorus concentration in extracellular fluid.

**Fig. 9:**
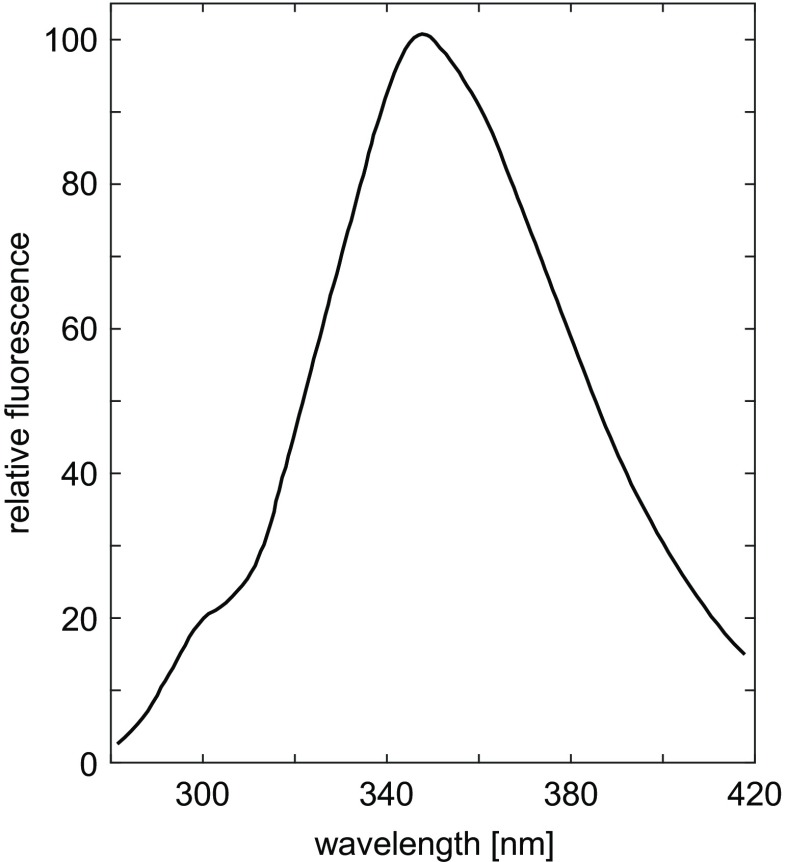
Fluorescence spectrum of PTH, pH 6.1, 0.08M KCl, 0.02M lysine, with excitation at 270 nm. Adapted from Edelhoch et al. (1969)

Bovine PTH (BPTH) is a single polypeptide chain of 83 amino acids and only one Trp and one Tyr (Edelhoch and Lippoldt 1969). The emission spectrum of BPTH at pH 6.1 (excitation at 270 nm) is depicted in Fig. 9. The fluorescence of Tyr is visible as a pronounced shoulder at 300 nm. The peak of Trp fluorescence in this polypeptide occurs at 347 nm. Position of the peak and the relative quantum yields of the two chromophores, make it possible to detect a distinctive band due to Tyr emission (Edelhoch et al. 1969). When BPTH is excited at 290, instead of 270 nm, 65 % of the Tyr, but only 10 % of the Trp emission is lost (Edelhoch et al. 1969).

The emission spectrum of BPTH in the range of 280 to 400 nm is almost superimposable on the emission spectrum of Trp-Gly_4_-Tyr (Edelhoch and Lippoldt 1969), suggesting that the average distance distribution between the two chromophores is similar in both peptides (Edelhoch et al. 1969). A more precise estimate of the distance between the two chromophores can be determined from the extent of Trp fluorescence quenching resulting from Tyr ionization.

**Fig. 10:**
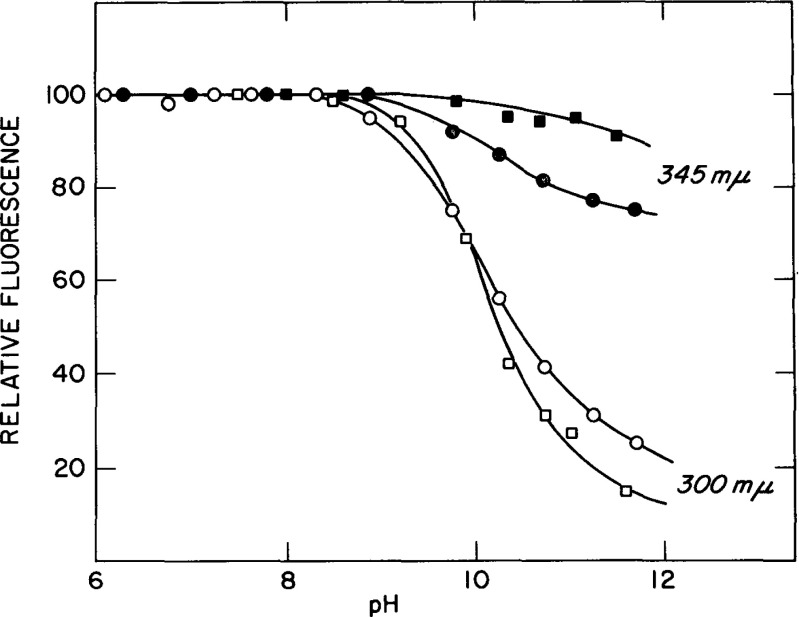
The alkaline dependence of tyrosyl (300 nm) and tryptophan (345 nm) fluorescence of PTH in aqueous solution (*circles*) 0.090M KCl, 0.02M lysine and in 4.6 M guanidine-HCl (*squares*). Excitation at 270 nm at 25 ^∘^C. Taken from Edelhoch et al. (1969)

The quenching of Trp fluorescence emission by radiationless energy transfer (FRET) to ionized Tyr has been shown to be common in proteins in the alkaline pH range (Edelhoch and Lippoldt 1969; Steiner and Edelhoch 1963). Figure 10 shows the effect of pH on fluorescence of Tyr and Trp in BPTH. Both emissions are normalized to the same value at neutral pH. It can be noted that Trp fluorescence is quenched by 30 % if the curve is extrapolated to 100 % ionization of the Tyr. A proportionality between Trp quenching and Tyr quenching, holding in the full pH range of Tyr ionization, suggests that the quenching of Trp fluorescence is due solely to energy transfer to the ionized Tyr residue (Edelhoch and Lippoldt 1969). A much smaller degree of Trp quenching (10 %) of PTH by ionized Tyr is observed in 4.6 M guanidine solution (see Fig. 10). These results can be interpreted based on experiments with Trp-Gly$_{0\leftrightarrow 4}$-Tyr, which showed that the degree of quenching of Trp emission by Tyr ionization decreased regularly with increasing chain length, and fell to about 40 % in Trp-Gly_4_-Tyr (Edelhoch and Lippoldt 1969). Judging from the decrease in quenching efficiency in the series Trp-Gly$_{0\leftrightarrow 4}$-Tyr, the inter-residue distance in BPTH in guanidine-HCl is presumably greater than in water (Edelhoch et al. 1969; Edelhoch and Lippoldt 1969).

Insulin is a tryptophan-free peptide hormone produced by *β*-cells in the pancreas. It is essential for regulating homeostasis of blood glucose levels (Hua 2010), and used to treat insulin-dependent diabetics. It is a globular protein composed of two peptide chains, A and B, with 21 and 30 amino acid residues, respectively. The molecule is linked by three disulphide bridges (two inter-chain: A7-B7 and A20-B19, and one intra-A-chain: A6-A11). Four of the residues are Tyr: A14, A19, B16, and B26 and three are Phe: B1, B24, and B25.

Bekard and Dunstan (2009) monitored intrinsic Tyr fluorescence of bovine insulin in 0.1 % (v/v) HCl, to investigate its partial unfolding and fibrillation. The pH of solution (1.9), being well below the pK*a* of both ground state (pK_*a*_ = ∼10) and excited state (pK_*a*_ = ∼4.2) of the chromophore, ensured that deprotonation of the hydroxyl group and subsequent formation of tyrosinate or Tyr-carboxylate hydrogen bonds could be excluded. Fluorescence of Tyr residues in insulin was monitored using excitation of 276 nm, with emission measured at 303/305 nm for kinetic studies, or scanned in the interval 280-500 nm for equilibrium studies.

**Fig. 11:**
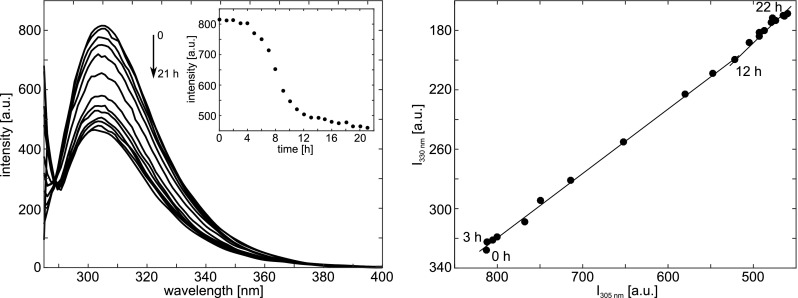
*Left:* Fluorescence emission spectra of Tyr during insulin aggregation initiated by incubation under conditions favoring fibrillation. Spectra registered at 1-h intervals are shown. The *inset* shows a sigmoidal change observed in the emission intensity at 305 nm as a function of time; *Right:* Phase diagram obtained from Tyr fluorescence data that reveal the existence of structural intermediates on thermal denaturation of insulin. Taken from Bekard and Dunstan (2009)

Figure 11 (left) presents the Tyr fluorescence spectra of insulin incubated under conditions favoring fibrillation as a function of time and shows a decrease in emission intensity with time. Simultaneously, λ_*max*_ of the band is constant at 305 nm. Plotting the emission intensity at 305 nm against time (shown in the inset) gives a sigmoid curve. Taken together, data presented in Fig. 11 (left) clearly shows a fast decrease in emission intensity after ∼2 h incubation, and reaching quasi-equilibrium state at 12 h. The authors discussed several possible catalysts for the observed Tyr fluorescence quenching preceding insulin fibrillation (Bekard and Dunstan 2009).

Further insight into the mechanism of insulin fibrillation is provided by a phase diagram of Tyr fluorescence intensity at 330 and 303 nm (Fig. 11 right). The phase diagram method consists of building up a correlation between two fluorescence intensities (extensive parameters in general) at two wavelengths, *I*(λ_1_) and *I*(λ_2_), under different experimental conditions, thereby forcing the protein to undergo structural transformations (Ahmad et al. 2003). When applied to protein folding, the relation *I*(λ_1_) = *f*[*I*(λ_2_)] is linear if changes in the protein environment lead to a two-state transition between two different conformations. The presence of a number of linear segments indicates the sequential structural transformations, where each linear portion of the *I*(λ_1_) = *f*[*I*(λ_2_)] plot describes an individual all-or-none transition (Ahmad et al. 2003).

Bekard and Dunstan (2009) applied phase diagram analysis to a study of the effect of temperature on insulin between 10 and 95 ^∘^C. The resulting fluorescence phase plot (Fig. 11 right), exhibits three linear segments suggesting two intermediate states in the transition from native to provisionally denatured insulin.

VanderMeulen and Govindjee (1977) suggested the molecular mechanism of photophosphorylation in chloroplasts. They measured Tyr fluorescence polarization of ATP synthase, excited at 280 nm in the presence of cations, ADP and P_*i*_. ATP synthase is an enzyme that provides energy by synthesizing adenosine triphosphate (ATP).

**Fig. 12:**
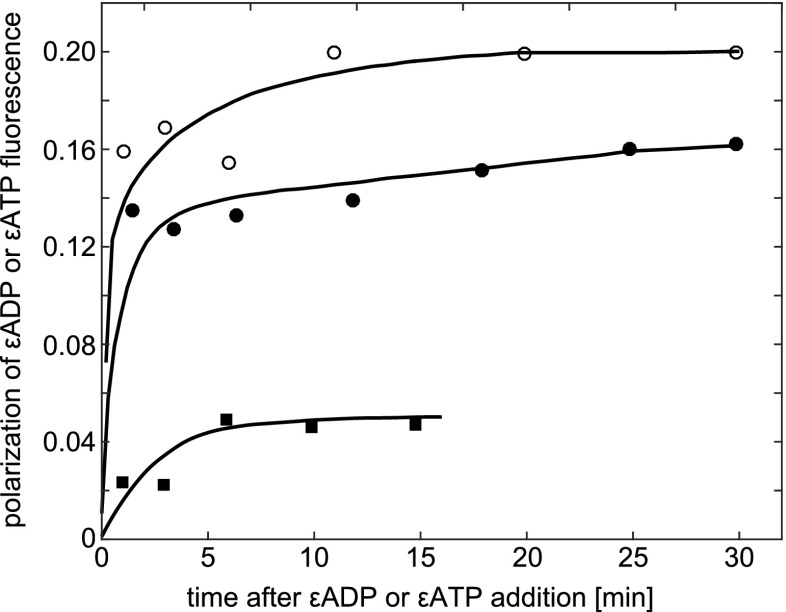
The effect of various salts on polarization of Tyr fluorescence in ATP synthase. The medium contained 25 mM tricine-NaOH (pH 8.2) and 3.6 *μ*M protein. Taken from VanderMeulen and Govindjee (1977)

Figure 12 shows the polarization data for Tyr fluorescence in ATP synthase to monitor possible functional changes in its conformation induced by substrates or cofactors of phosphorylation. The addition of divalent cations results in a 20 % increase in polarization of Tyr fluorescence. MgCl_2_ is somewhat more efficient than CaCl_2_ in producing this effect at low (2.5-20 mM) concentrations but this disappears at 30 mM. With KCl, the resulting changes are much smaller in the same concentration range. The addition of 2.5-20 mM MgCl_2_ had little or no effect on either the UV absorption (280 nm) or fluorescence spectra, suggesting that the observed salt-induced changes in polarization of ATP synthase fluorescence are not due merely to a decrease in the lifetime of Tyr fluorescence. The authors suggest these changes are due to an altered protein structure in which, for example, the mobility of one or more Tyr residues is reduced.

Our next example is a study of fluorescence polarization decay of Tyr in lima bean trypsin inhibitor (LBTI), an 83 amino acid protein with seven disulphide bonds (Nordlund et al. 1986). Trypsin inhibitor is a serine protease inhibitor that reduces the biological activity of trypsin, an enzyme involved in the hydrolysis of proteins during digestion.

Nordlund et al. (1986) investigated the fluorescence anisotropy decay of the single Tyr-69 in LBTI to gain insight into the time-scale of structural fluctuations and motions of this protein. Tyr fluorescence lifetime, obtained from a single-exponential, is 620 ±50 ps. The results of anisotropy decay are interpreted by comparison with the anisotropy decay of N-Ac-Tyr-NH_2_ in a viscous medium. In each case, experimental results were fitted to

13 $$ r(t) = r_{0} \mathrm{E}^{-t/\phi} \left[a\mathrm{e}^{-t/\phi_{i}} + (1-a)\right], $$

where *ϕ* is the rotational correlation time for the whole protein, *ϕ*_*i*_ is that for the internal motion, and *a* is the fraction of the total anisotropy that decays as a result of the internal motion. Assuming time zero as the time corresponding to the center of the excitation pulse, Norden et al. fit the fluorescence anisotropy decay of LBTI to a dual-exponential form, with time constants of about 40 ps and 3 ns. The nanosecond component is consistent with rotation of the entire protein molecule. The 40-ps component demonstrates that the Tyr has much greater freedom of motion.

## Structural analysis of proteins measured by linear dichroism of tyrosine residues

LD provides information about the average orientation of the Tyr residues in proteins. Here we review a study of the Rad51-DNA filament (Reymer et al. 2009), a homologue of Escherichia coli RecA. Rad51 is a protein involved in repair of double-strand breaks in DNA in eukaryotic cells (Baumann and West 1998). Rad51 and RecA form extended filaments on DNA and promote the DNA strand exchange in recombinant DNA repair (Cox 2009). Combining molecular modeling with LD of the human Rad51-dsDNA-ATP complex in solution (HsRad51-dsDNA-ATP), Nordén et al. (Reymer et al. 2009) proposed a model corresponding to the final product of the recombination reaction. Their experimental approach, called site-specific linear dichroism (SSLD), is based on molecular replacement of individual aromatic residues in the protein with other aromatic residues, providing angular orientations of the replaced residues relative to the filament axis.

The HsRad51 lacks Trp, but contains 10 Tyr residues that were replaced, one at a time, by Phe, using site-directed mutagenesis. The Tyr to Phe modification corresponds to a structural elimination of an oxygen atom, and is expected to leave the protein structure unchanged. This was confirmed by comparing CD spectra of the wild-type (WT) and mutated proteins. The difference LD spectrum (SSLD) between the WT and modified proteins, was considered to represent the LD spectrum of the replaced residue itself, after appropriate normalization for extinction coefficients of Tyr and Phe, and correction for the background.

**Fig. 13:**
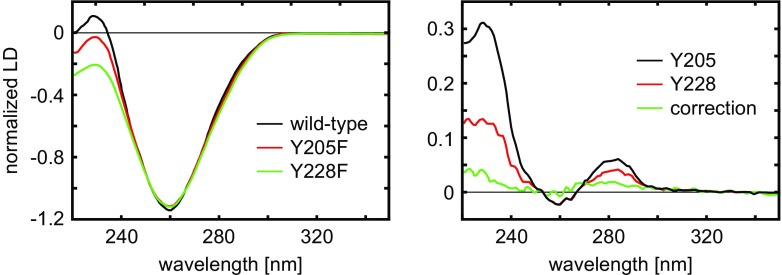
*Left:* Experimental flow LD spectra of wild-type and two selected modified HsRad51 nucleo-protein complexes; *Right:* resulting SSLD spectra for the corresponding Tyr residues. Adapted from Reymer et al. (2009)

Figure 13 left presents experimental flow LD spectra of the WT HsRad51-DNA-ATP filaments and two modified nucleoprotein filaments, in which Tyr was replaced by Phe at residues 205 and 228. Figure 13 right shows two SSLD spectra for corresponding Tyr residues and the baseline correction spectrum.

There are distinct variations in the spectra of modified protein complexes, compared to WT protein alone. The most obvious differences are centered at 230 nm, but the spectra also differ around 280 nm. These wavelengths correspond to the L_*a*_ and L_*b*_ transition moments, respectively, of Tyr.

Assuming that the structures of nucleo-protein filaments formed by WT and modified proteins are comparable, the SSLD spectrum of the particular substituted residue can be computed as the differential spectrum of the LD spectrum of the WT and mutated protein complexes. The SSLD spectrum of the substituted residue may be used to determine orientation coordinates of the replaced residue chromophore with respect to the filament axis oriented along flow lines of the solvent in the flow LD experiment.

**Fig. 14:**
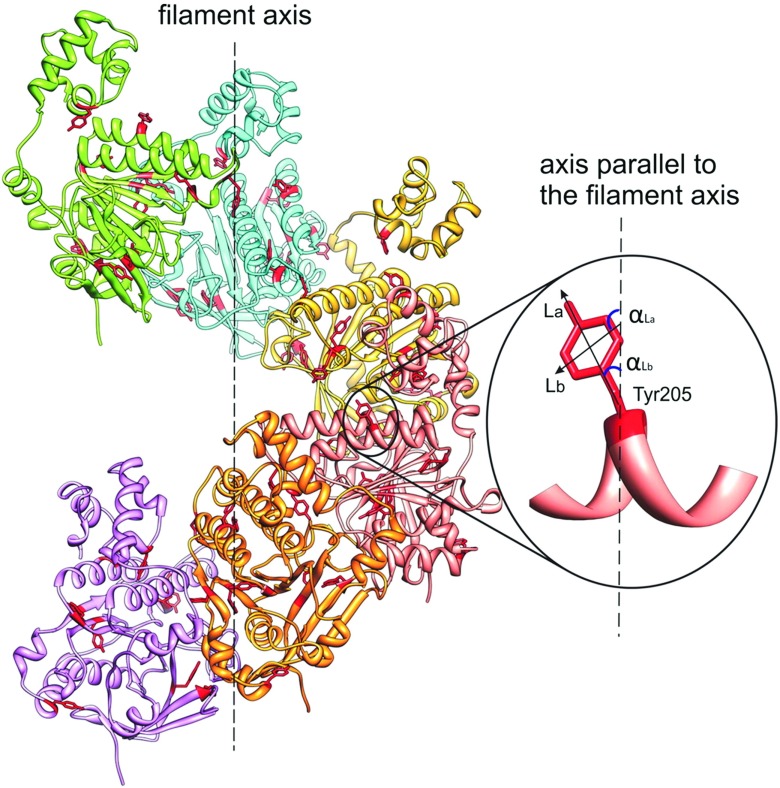
Molecular model of the HsRad51 helical filament showing the angular orientations of transition moments L_*a*_ and L_*b*_ of Tyr-205 residue, relative to the filament axis. Taken from Reymer et al. (Reymer et al. 2009)

The angular orientation *β* of the transition moments, for each substituted Tyr residue relative to the helix axis of the nucleo-protein filament, can be determined from the reduced LD if the orientation factor S in Eq. 6 of Part 1 is known. S is the degree of orientation of the filamentous complex achieved in the flow LD experiment. The value of this parameter was computed by Nordén et al. (Reymer et al. 2009), assuming that the orientation of DNA in the complex with Rad51 is similar to that in the RecA-DNA complexes assessed from small-angle neutron scattering (Nordén et al. 1992). Using this approach, the orientation angles for eight of the ten Tyr residues of HsRad51 were determined from the SSLD spectra. Two angular orientations, the L_*a*_ and L_*b*_ transition moments, were reported for each Tyr. The meaning of these orientations is explained by referring to Fig. 14. For Tyr205 and Tyr228 (the SSLD spectra are shown in Fig. 13) the L_*a*_ and L_*b*_ angles are 43 ^∘^ and 41 ^∘^, and 28 ^∘^ and 30 ^∘^, respectively.

The published results (Reymer et al. 2009) were discussed in a later publication (Fornander et al. 2014) in the context of the evidence that the spectral shifts of the Tyr chromophore, *p*-cresol, are due to environmental changes, mainly a result of its ability to form hydrogen bonds. The observed clear red- (Tyr54 and Tyr216 of HsRad51) and blue shifts (Tyr205 and Tyr228) of the absorption bands of L_*a*_ transition were interpreted (Fornander et al. 2014) by referring to the hydrogen bonding capabilities suggested by considering previously developed fragments of the Rad51 protein structure based on crystallography and NMR (Aihara et al. 1999; Pellegrini et al. 2002; Conway et al. 2004).

## Tyrosines in near-UV CD spectrometry of proteins

Tyrosyl CD bands of proteins in the near-ultraviolet may be used to study their tertiary structure in solution. However, this is complicated by Trp, Phe, and Cys residues absorption bands in the same wavelength range. Horwitz et al. (1970). Even in proteins lacking Trp, the Tyr CD bands are not easily identified, nor can their intensities be accurately assessed, because of ambiguity due to disulphide contributions. One instructive example is given by ribonuclease A (RNase-A) where the 275-nm CD band can be mostly attributed to Tyr. Conflicting interpretations depend on whether buried or exposed Tyr side chains can generate the observed CD band (Horwitz et al. 1970; Simpson and Vallee 1966; Simmons and Glazer 1967).

**Fig. 15:**
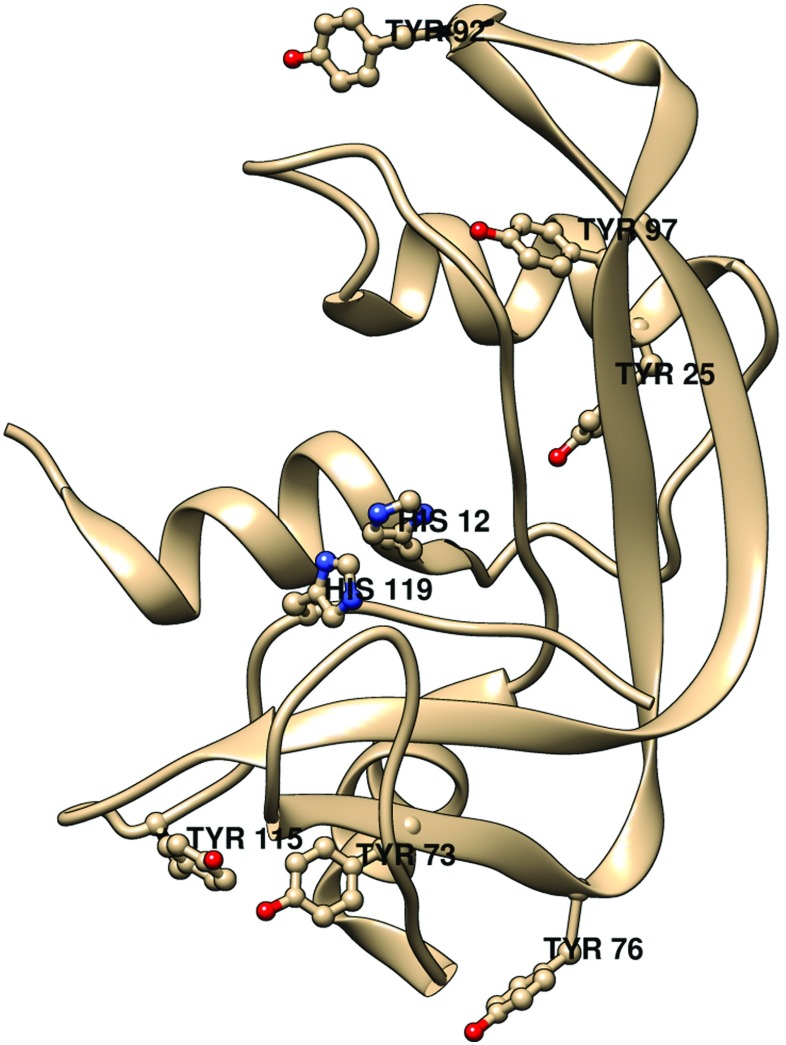
A ribbon diagram of bovine pancreas RNase-A showing the six tyrosines. His12 and His119 are also shown to indicate the location of the active site. Generated by USCF Chimera [REF: Pettersen EF, Goddard TD, Huang CC, Couch GS, Greenblatt DM, Meng EC, Ferrin TE (2004) J Comput Chem 25:1605] from the structure of RNase A by Howlin et al. (1989).

Bovine pancreatic RNase-A contains six Tyr residues, three Phe residues, no Trp, and four conserved disulphide bonds (Howlin et al. 1989; Chatani et al. 2002) (see Fig. 15). Three RNase-A tyrosines, Tyr25, Tyr92, and Tyr97, are buried and three, Tyr73, Tyr76, and Tyr115, are exposed to solvent.

Using absorption and CD spectra of tyrosine and three of its derivative, registered at 298 and 77 ^∘^K, and referring to the vibronic transitions of *p*-cresol, Horwitz et al. (1970) analyzed absorption and CD spectra of RNase-A, also at 298 and 77 ^∘^K. They intended to determine the relative contributions of the various types of Tyr residues. They identified 0-0 transition for two tyrosines at 286 nm, for one tyrosine at 289 nm, and for three tyrosines at 283 nm, in the absorption spectrum at 77 ^∘^K. Spectral positions of the latter indicated they are solvent-exposed. In the CD spectrum of RNase-A, obtained at 77 ^∘^K, they identified well-resolved bands at 282 and 276 nm, and shoulders at 288.5, 267.5, 261, and 255 nm. The first three were assigned to Tyr, and the last three to Phe. Horwitz et al. (1970) found no CD band at 286 nm, corresponding to two Tyr residues with 0-0 absorption at this wavelength. They concluded that the contribution from these Tyr residues is insignificant.

Recently, much more extensive information on Tyr side-chain contributions to the spectra (both in the near- and far-UV range) of RNase-A was presented by Woody and Woody (2003). They measured and computed CD spectra of wild-type (WT) and six Tyr to Phe mutants. Comparison of WT and mutated RNase-A CD spectra, both experimental and theoretical, make it possible to resolve roles of individual Tyr to the CD spectra. Here we limit the discussion to experimental CD spectra in the near-UV range.

Comparing the far-UV CD spectra of the Tyr →Phe mutants with the spectrum of the WT protein, at 2 ^∘^C and pH 7, Woody and Woody (2003) concluded that the secondary structures of the mutants are not significantly perturbed. Thermal unfolding experiments of WT RNase-A and the mutants revealed that melting temperatures of the mutants vary only slightly from that of the WT. The largest deviations, [T_*m*_(mutant) −*T*_*m*_(WT)], were observed for two of the mutations at buried Tyr sites: −5.0 ^∘^C for Y25F, and −9.7 ^∘^C for Y97F.

**Fig. 16:**
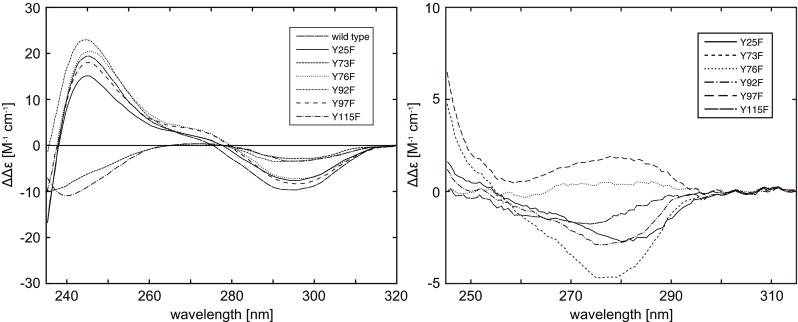
*Left:* The pH difference CD spectra (pH 11.3 and 7.0, 2 ^∘^C) of RNase A wild-type and Tyr →Phe mutants.; *Right:* Experimental near-UV CD difference spectra (2 ^∘^C) of RNase-A wild-type minus the mutants at pH 7.0. Taken from Woody and Woody (2003)

Three exposed Tyr in RNase-A titrate with a pK_*a*_ of ∼9.9 and three buried tyrosines have elevated pK_*a*_ values (Shugar 1952; Tanford et al. 1956), thus Woody and Woody expected to observe a difference in the CD spectra due to contributions of the three exposed and three buried Tyr by raising the pH. The pH difference spectra of the mutants and the WT RNAse are shown in Fig. 16 left. The difference spectrum for the WT has a negative band at 295 nm and an enhanced positive band at 245 nm. The mutants of the three buried Tyr (Y25F, Y92F, and Y97F) and one exposed Tyr (Y76F) all exhibit pH difference spectra similar to the WT. The mutants of two exposed Tyr (Y73F and Y115F) exhibit diminished 295-nm negative bands and, instead of positive bands, negative bands are observed at 245 nm. Assuming that disulphide and peptide bond contributions to the CD spectra do not change substantially with changing pH, the dual differences in the ellipticities ΔΔ*𝜖*_295_≡Δ*𝜖*_295_(pH 11)−Δ*𝜖*_295_(pH 7) and similarly defined ΔΔ*𝜖*_245_ probably represent contributions from Tyr alone.

Analyzing the values of ΔΔ*𝜖*_295_ and ΔΔ*𝜖*_245_, Woody and Woody noted that mutation of two of the buried Tyr (Y25F and Y97F) and one of the exposed Tyr (Y76F) slightly perturb the ΔΔ*𝜖*_295_ and ΔΔ*𝜖*_245_ values from those of the WT. This suggests that the contributions of these Tyr to the 277- and 240-nm bands are small, but not negligible. Two subsequent mutants (Y73F and Y115F) show a substantial reduction in the ΔΔ*𝜖*_295_ values and exhibit negative ΔΔ*𝜖*_245_ values, understood as indicating that these two exposed Tyr are a significant source of the 277- and 240-nm bands.

The right panel in Fig. 16 shows the Tyr contributions to 277-nm CD difference spectra for WT and the mutants. The mutants at two of the exposed Tyr sites (Y73F and Y115F) have reduced negative intensity in the near-UV CD band. This results in more negative ΔΔ*𝜖*_277_≡Δ*𝜖*_277_(WT)−Δ*𝜖*_277_(mutant) values than those from Y25F and Y92F. The mutants Y76F and Y97F show positive ΔΔ*𝜖*_277_ values. These spectra indicate not only that the two exposed Tyr (73 and 115) contribute significantly to the CD at 277 nm, but also that the contribution of buried Tyr are not negligible. Another interesting result is the ΔΔ*𝜖*_286_ (defined in analogy to ΔΔ*𝜖*_277_) value for the WT-Y92F is negative and the value for the WT-Y97F is positive. Thus, the near-UV CD contributions of Tyr92 and Tyr97, two buried tyrosines, are comparable in magnitude, but opposite in sign. According to Woody and Woody (2003), this explains why Horwitz et al. (1970) observed no CD band at 286. It was a consequence of the canceling contributions of Tyr92 and Tyr97.

## Resonance Raman scattering from tyrosine residues

We now present several applications where the determination of the position and relative intensities of a closely spaced pair of Raman lines at ∼850 and ∼830 cm ^−1^ due to Tyr is used. This is an example of a more general phenomenon known as the Fermi resonance doublet (Larkin 2011).

Using continuous-wave excitation at 244 nm, Couling et al. (1997) utilized the intensity ratio, *R*_244_ = *I*_834_/*I*_855_ to investigate the environment of Tyr residues in native and denatured barnase. Barnase is a bacterial ribonuclease, consisting of 110 amino acids. It has three Trp (35, 71, 94) and seven Tyr (13, 17, 24, 78, 90, 97, 103) residues (Fersht 1993). In the denatured form, all seven Tyr residues are exposed to the solvent. In the folded state, two, Tyr13 and Tyr17, remain exposed to solvent, while the other five are buried in hydrophobic cores (Matouschek et al. 1992).

**Fig. 17:**
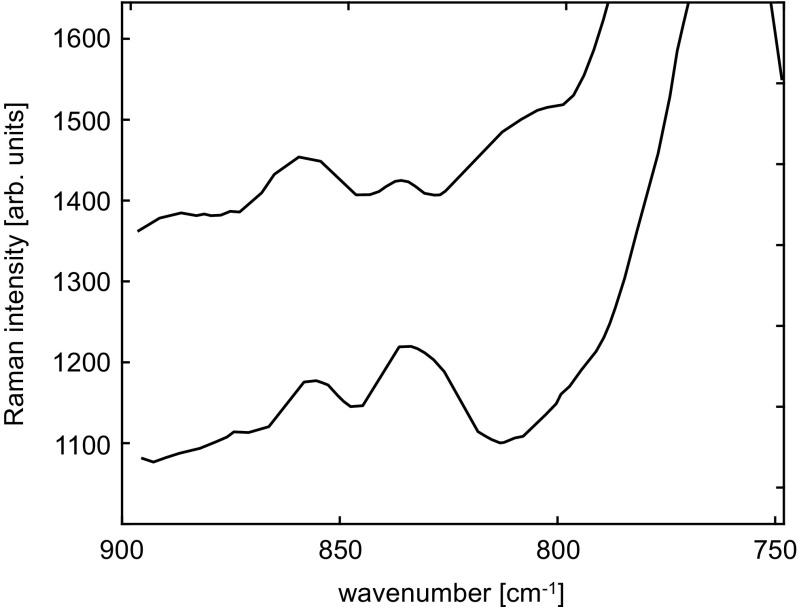
Resonance Raman spectra of barnase in the range 750-890 cm ^−1^ obtained with excitation wavelength 244 nm, for denatured wild-type barnase (pH 1.5, *top*) and folded wild-type barnase (pH 6.3, *bottom*). The spectra have been displaced for clarity. Adapted from Couling et al. (1997)

Figure 17 shows Fermi-resonance doublets for denatured and folded wild-type barnase. In principle, one expects resonance Raman signals from both the Trp and Tyr residues. Furthermore, these features will be the average for the three Trp and seven Tyr residues. The Fermi-resonance doublet seen at 834/855 cm ^−1^ is the average of all seven Tyr, so care is needed if the change in intensity ratio, *R*_244_ = *I*_830_/*I*_850_, of the doublet is to yield quantitative information. In the denatured state, all seven Tyr residues are solvent-exposed, so the *R*_244_, from intensities in the Figure, is *R*_244_(denatured)=0.67±0.09, whereas for folded barnase,

$$R_{\mathrm{244}}(\text{folded}) = \mathrm{1.60\pm 0.16} = \frac{2}{7}\cdot \mathrm{0.67} + \frac{5}{7}\cdot R_{\mathrm{244}}(\text{buried}) $$

which yields 1.97 as the ratio for buried Tyr residues. A general formula for *R*_244_ to be observed in barnase with mole fraction *x*_*e*_ of exposed Tyr and mole fraction *x*_*b*_ of buried Tyr is thus

$$R_{\mathrm{244}} = 1.97\cdot x_{b} + 0.67 \cdot x_{e} \qquad \text{with} \qquad x_{e}+x_{b}=1$$

Subsequently, one can use the Couling et al. (1997) linear relation between *R*_244_ and enthalpy of hydrogen bond formation Δ*H*, obtained from values of the Tyr Fermi-doublet intensity ratio, *R*_244_ = *I*_830_/*I*_850_, for the model compound *p*-cresol in solvents of varying H-bond acceptor strengths,

$$R_{\mathrm{244}} = 0.31 \cdot \left( -{\Delta} H\right) + 0.19$$

where Δ*H* is in units of kcal ⋅*mol* ^−1^. Accordingly, one may estimate average enthalpies of hydrogen bonding for exposed and buried Tyr residues in barnase. From *R*_244_(exposed)=0.67, one gets Δ*H* = −1.55 kcal ⋅*mol* ^−1^, whereas from *R*_244_(buried)=1.97, one gets Δ*H* = −5.74 kcal ⋅*mol* ^−1^.

A second example is determination of the pK_*a*_ of Tyr in an 11-residue peptide fragment from transthyretin by Pieridou and Hayes (2009). Transthyretin (TTR) is a highly conserved homotetrameric protein, synthesized mainly by the liver and the choroid plexus of brain (Vieira and Saraiv 2014). It is a serum and cerebrospinal fluid carrier of the thyroid hormone thyroxine and retinol-binding protein bound to retinol. Originally, TTR was called prealbumin because it runs faster than albumin on electrophoresis gels.

The investigated 11-residue fragment has the sequence Tyr-Thr-Ile-Ala-Ala-Leu-Leu-Ser-Pro-Tyr-Ser, which is located at positions 105 to 115 along the main chain of TTR, TTR(105-115). This peptide fragment was shown to form amyloid fibrils, and hence may serve as a model system for studies of fibril formation in general (Gustavsson et al. 1991).

**Fig. 18:**
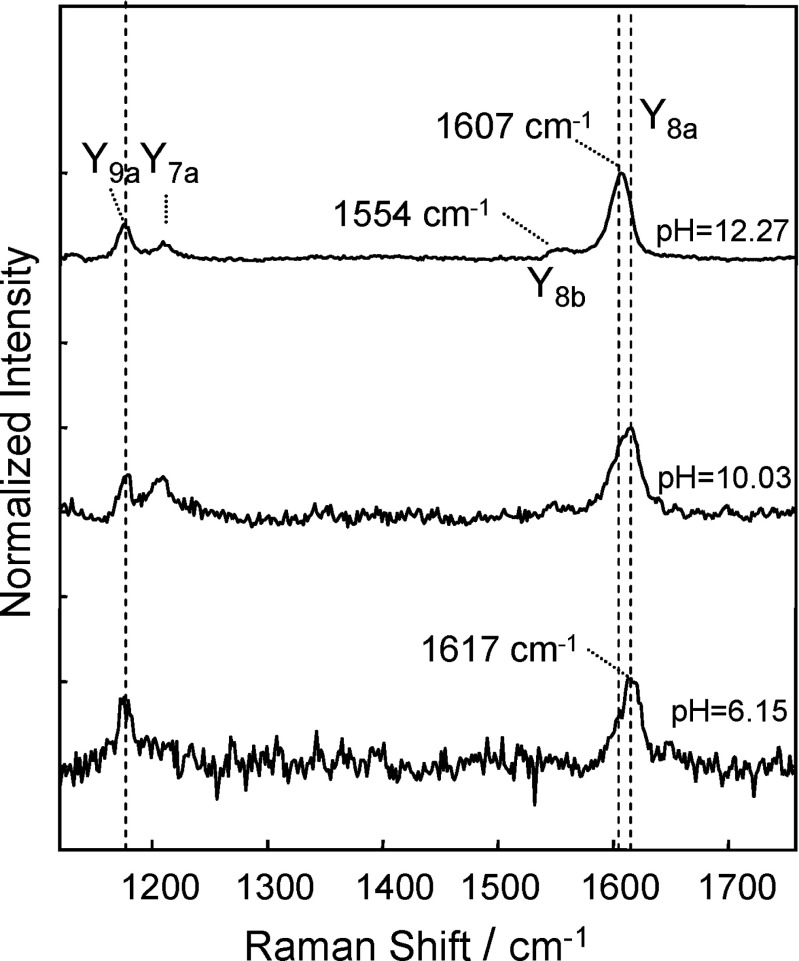
Resonance Raman spectra of TTR(105-115), in the wave-number range corresponding to vibrations of the phenolic side-chain of the tyrosines, excited at 239.5 nm, for three selected values of pH: 6.15, 10.03, and 12.27. Taken from Pieridou and Hayes (2009)

Pieridou and Hayes (2009) measured resonance Raman spectra of the TTR(105-115) peptide in solution as a function of pH, using excitation at 239.5 nm, which is resonant with the L_*a*_ excited electronic state of Tyr. Three examples of the spectra are shown in Fig. 18. At the excitation wavelength (239.5 nm), vibrational features are observed due solely to resonance enhancement of bands associated with the phenolic side-chain of the Tyr in the peptide. The changes in the spectra at alkaline pH indicate formation of tyrosinate from Tyr. In titration experiments, the band observed at 1617 cm ^−1^ at pH 6.15, Y_8*a*_ (where 8a refers to Wilson’s notation (Wilson 1934) clearly shifts with changing pH (to 1607 cm ^−1^ at pH 12.27).

Plotting the frequency of the Y_8*a*_ band as a function of pH produces the titration curve. Fitting the pH dependence to the Henderson–Hasselbach curve resulted in a pK_*a*_ of 10.2 ±0.2 for the peptide. This value represents an average value for the two Tyr in the peptide. The same method for free Tyr in solution gave 9.1 ±0.2.

Attempting to explain the higher pK_*a*_ value for the TTR(105-115) peptide relative to free Tyr, Pieridou and Hayes (2009) referred to microenvironments of the Tyr side-chains formed by the TTR(105-115) peptide. Tyr-105 occupies the N-terminal position and is expected to be fully exposed to solvent, hence its pK_*a*_ should be similar to that of free Tyr. Tyr-114, on the other hand, is amidst residues with H-bonding side-chains, Ser112 and Ser115, which can act either as proton donors or acceptors. In addition, the neighboring Pro-113 residue may maintain some specific structure within the peptide fragment. Both of these factors may elevate the pK_*a*_ of Tyr-114, and as a consequence the average pK_*a*_ value determined in the experiment is also elevated.

## Conclusions and perspectives

We have discussed absorption of UV light by the side-chain of Tyr residues in proteins, as well as several other spectroscopic properties of this chromophore, all valuable probes of protein structure.

Although large extinction coefficient, emission quantum yield, and high sensitivity to changes in the microenvironment have led to Trp being routinely used as an intrinsic marker in proteins, inclusion of Tyr in the arsenal of chromophores used for characterization of the protein structure has clear advantages. Phenol is more polar than indole, therefore Tyr should react more strongly to environmental changes than Trp. Shifts in the absorbance spectrum due to changes in the polarity of the environment are generally larger for Tyr than for tryptophan (Fornander et al. 2014).

The phenol motif in Tyr provides unique physicochemical properties and chemical reactivity that enables this amino acid residue to achieve a plethora of biosynthetic transformations and molecular interactions (Jones et al. 2014).

The spectroscopic properties described above may be used to follow these transformations and gain insight into these interactions. Experiments described in this review may present useful hints for studies of structural properties of proteins, employing other possible experimental and/or theoretical methods because UV–Vis absorption and fluorescence spectrometers, as well as their more specific developments of LD, CD, or resonance Raman scattering, are readily available, and it is easy to supplement an investigation with these spectroscopies.
